# A study on the effect of the number of expansion units in a microfluidic chip on hyaluronidase-free oocyte denudation in mammals

**DOI:** 10.2478/joeb-2025-0004

**Published:** 2025-03-20

**Authors:** Ashraf Hisham Dessouky, Haitham EL-Hussieny, Taymour Mohammed EL-Sherry, Victor Parque, Ahmed M. R. Fath El-Bab

**Affiliations:** Department of Mechatronics and Robotics Engineering, Egypt-Japan University of Science and Technology (E-JUST), Alexandria 21934, Egypt; Mechanical Engineering Department, Helwan University, Cairo 11792, Egypt; Department of Theriogenology, Veterinary Hospital, Faculty of Veterinary Medicine Assiut University, Egypt; Graduate School of Advanced Science and Engineering, Hiroshima University, 1-4-1 Kagamiyama, HigashiHiroshima 739-8527 Japan

**Keywords:** Microfluidic, oocyte denudation, CO_2_ laser, in vitro fertilization, expansion units

## Abstract

In Vitro Fertilization (IVF) and Intracytoplasmic Sperm Injection (ICSI) are well-known fertility treatments that, due to resource-intensive, high degree of expertise required, and frequent subpar performances, often yield in high costs for treatment cycles. Microfluidic technology has enabled cost-effective egg-handling procedures towards new assistive reproductive devices: oocytes are subjected to microchannels with jagged surfaces to let shear stress remove undesirable cumulus cells, and microchannels with expansion units facilitate the transport of oocytes in chips. However, although the previous works have studied the influence of shear stress on oocyte denudation and the role of microchannel teeth in optimizing cell handling efficiency, the study of configurations of jagged surfaces and expansion units in microfluidic devices has remained elusive. Also, comprehensive analysis using both computational fluid dynamics (CFD) and real-world microfluidic devices has remained an unexplored area. To fill the abovementioned gap, this paper studies microfluidics chips with different expansion units to depict the behavior of oocytes when subjected to controlled input flows. The proposed chips were developed and fabricated using a direct engraving CO_2_ laser machine on polymethyl methacrylate (PMMA) sheets and bonded in a natural ventilation lab oven, rendering the highly efficient and low-cost microfluidic chips for oocyte denudation. The effect of the expansion units has been investigated in CFD simulation and real lab experimentation with mature buffalo oocytes at a constant flow rate, and a chip with five expansion units arranged in two lines achieved 98.33% denudation efficiency, low-cost fabrication (about 1 USD), and quick fabrication time (about 20 minutes).

## Introduction

Research in recent years has associated lower lifestyle scores with infertility [1]. As such, reproductive health can often be linked with anatomical, emotional, and behavioral changes such as increased appetite and fear [2,3]. Disruptions in reproductive processes can significantly impact a woman’s fertility and contribute to various reproductive system disorders, whose prevalence has risen in recent years [4,5]. Infertility is characterized as the inability to conceive a child after 12 months or more of uninterrupted, unprotected sexual intercourse [6]. The global prevalence of infertility among couples of reproductive ages varies between 12.6% and 17.5% in specific regions such as the Americas, Western Pacific, Africa, and Europe [7]. And, according to a report from the World Health Organization (WHO), 1 in 6 people are affected by infertility worldwide [8].

Assisted reproductive technology (ART) can be used to treat infertility, e.g., IVF and ICSI [9], and in-vitro oocyte maturation has shown excellent benefits such as reducing the health risks and diminishing ovarian hyperstimulation syndrome (OHSS) [10]. However, despite the widespread availability and the early success in diagnosing and treating infertility, contemporary ART techniques still face several drawbacks, such as high cost, limited accessibility, and social stigma, becoming unsuitable to patients [11]. The ART procedures have relied on the expertise of skilled individuals and, historically, have lacked the concomitant and required standardization, rendering results that are contingent upon operator skill [12].

To overcome the above-mentioned issues, microfluidics has become a relatively new field involving the study and manipulation of both fluids and micro/nanoparticles in microchannels often manufactured on tiny surfaces [13]. Microfluidics has demonstrated significant advantages in biomedical applications and research [14], especially in ART. Fertilization through microfluidics has been an area of intensive research in pigs and mice when oocytes were subjected to medium flow and semen through small tubes [15,16,17]. Researchers can also capture restricted-sized functional samples using microfluidic chips, which only need a minimal number of cells [18].

For successful IVF and ICSI, essential processes are essential: sperm sorting, to select the excellent quality, and oocyte denudation, to remove the cumulus cells around the oocyte [19,20]. By forming gap junctions with the oolemma, the cumulus cells often extend the cytoplasmic projections beyond the zona pellucida [21]. In IVF clinics and reproductive procedures, oocytes require denudation from cumulus cells to enable fertilization; this is achieved by immersing the oocyte in the enzymatic action of hyaluronidase and mechanical pipetting to separate cumulus cells from oocytes [22,23]. Hyaluronan comprises repeated disaccharide units of N-acetyl-D-glucosamine and D-glucuronic acid [24]. One of the components of cumulusoocyte complexes is hyaluronan, which is highly hydrated and viscoelastic [25]. Hyaluronidase treatment causes cumulus cells to disperse from oocytes and the breakdown of the hyaluronan-based matrix surrounding the cumulus cell-oocyte complex (COCs) [26,27]. Human oocytes treated with hyaluronidases had a lower survival rate, fertilization rate, and post-ICSI development rate than untreated oocytes [28,29,30]. Also, after being treated with hyaluronidase, the fertilization rate of the mouse oocyte dropped. The ability of oocytes to progress to the morula and blastocyst stages was also diminished after being treated with hyaluronidase for 5 minutes or more [31].

The oocyte denudation aims to facilitate the fertilization process by enhancing the injection of sperm into the oocyte [32]. Zeringue et al. manually removed the cumulus cells from individual bovine zygotes (the first stage of the early embryo) [33,34] using a microfluidic system. The proposed device underwent partial removal and reorientation of the cumulus cell mass. Following oocyte denudation, the cumulus-zygote complex passed through a channel being a little wider than the complex itself. Then, the remaining corona cells were extracted from the oocyte using two suction ports smaller than the complex. Denudation of the cumulus-zygote complex requires a manual approach involving switching and regulating fluid flows to position and move one zygote at a time.

Weng et al. [35] have created a microfluidic system to eliminate cumulus cells from mouse oocytes. COCs treated with hyaluronidase traversed a series of irregularly surfaced microchannels. The cumulus cells were removed by the inner wall of the constriction channel during the processing of COCs via a minimum of 100 cycles of expansion and constriction units. The device described by Weng et al. requires intricate manufacturing and use of hyaluronidase, which results in high manufacturing costs and extensive fabrication time due to the use of PDMS. Still, the device inherits all drawbacks from the concomitant use of hyaluronidases. Despite the suitability for microfluidic chips, nontoxicity, biocompatibility, and good thermal stability [36], PDMS still faces drawbacks such as high fabrication costs, time-consuming processing, lower mechanical stability, and the need for specialized bonding techniques, making PMMA a more practical alternative for large-scale and cost-effective applications.

Chen et al. [37] implemented a multi-channel microfluidic chip with a newly designed microcolumn to capture and measure the penetration reaction of three oocytes under varying Cryoprotectant (CPA) concentrations, improving measurement efficiency significantly. Mokhtare et al. [38] created a microfluidic device for contactless oocyte denudation by reshaping surface acoustic waves (SAW). The device uses biocompatible microwells and four interdigitated transducers (IDTs) to create ultrasonic and remove cumulus cells, resulting in total denudation. Zhai et al. [39] developed a robotic system to automate the removal of mouse cumulus cells from oocytes. The system uses vision-based contact detection, oocyte and cumulus cell recognition, automatic calibration, and micropipette aspiration and deposition control, achieving a denudation efficiency of 95.0 ± 0.8%. Fang et al. [40] investigated the recent advances in microfluidic in-vitro fertilization technologies, showing that microfluidics can significantly enhance assisted reproductive technology (ART) by miniaturizing, integrating, and automating various stages of embryo production.

Oocyte denudation in microfluidic chips requires design and manufacturing of channels featuring jagged surfaces and expansion units. Also, realizing the effective and low-cost microfluidic chips are contingent upon materials that are readily and widely available on the market, being biocompatible, transparent, and with no sign of leakage post-bonding. The above-mentioned features are also essential to investigate cell behavior when subjected to distinct configurations of expansion units and jagged surfaces under low-cost and high-quality considerations. Polymethyl Methacrylate (PMMA) is used to manufacture microfluidic chips for several biological applications, including Deoxyribonucleic acid (DNA) analysis, polymerase chain reaction (PCR), and cell separation [41,42,43]. Microfluidic chips have been developed using PMMA for sperm sorting and optimization based on the rheotaxis approach [44,45]. Micro biosensors have been fabricated with PMMA material [46]. Furthermore, several researchers have previously investigated the micromachining of PMMA via a CO_2_ laser [47,48,49,50,51]. The direct engraving approach produced several microfluidic chip materials using a laser beam in several bio-applications [52,53,54]. Microfluidics has been used to explore the transport mechanism of Hg(II) [55] and evaluate the critical suitability of Caco-2 cells [56] as gut-on-a-chip.

Despite the advances in microfluidics to investigate cell behavior in fertilization fields, the study of configurations of oocyte behavior when subjected to distinct configurations of expansion units and jagged surfaces under low-cost and high-quality considerations has remained elusive. The comprehensive analysis utilizing both computational fluid dynamics (CFD) machinery and real-world fabricated microfluidic devices has remained an unexplored area in literature. To fill the above-mentioned gaps, this paper studies microfluidics chips with different expansion units to study the behavior of oocytes when subjected to controlled input flows. As such, it becomes possible to identify the suitable configuration to achieve the highest oocyte denudation performance at a low cost and with a quick fabrication time. Our contributions are as follows:

- Conducted CFD simulations to analyze the effect of wall shear stress on oocytes in different microfluidic chip designs with varying expansion unit lengths and flow rates.
- Experimentally validated the simulation results by fabricating the corresponding microfluidic chips using CO_2_ laser engraving.
- Performed real-world oocyte denudation experiments using buffalo oocytes to assess the effectiveness of the designed chips.
- Evaluated oocyte denudation performance based on the denudation success rate (efficiency) under different expansion unit configurations.

In the rest of the paper, we introduce the materials and methods and then discuss the results we obtained.

## Materials and methods

### Selecting the designs

The dimensions of microfluidic chips correspond to the conventional size often used in microscope slide format, measuring 75.5 × 25.5 mm. Microfluidic chips have been fabricated using 3 mm thick and 0.1 mm PMMA sheets. The chips include three PMMA layers: the upper layer is 3 mm thick and serves as the cover with input and output apertures, while the middle layer is 0.1 mm thick and thoroughly engraved with the necessary design, and the bottom layer is 3 mm thick and serves as a cover to close the microfluidic chip; hence, the overall height of the chip is 6.1 mm.

Oocyte denudation requires the removal of the cumulusoocyte complex matrix from the oocyte. Consequently, microfluidic channels need internal jagged surfaces with precise width and depth dimensions dependent upon the oocyte’s size. Typically, mammalian oocytes are round, with a diameter of 80 µm in mice and 120–130 µm in bigger animals like humans [57]. The average diameter of the buffalo oocytes removed from normal ovaries was 146.4 microns [58]. When accompanied by cumulus cells, the oocyte may be as large as 350 to 450 µm in diameter. Thus, the channel depth is constant due to the thickness of the middle layer. The inner jagged width design is 0.1 mm. The overall channel length is 62.76 mm.

**Fig.1:**
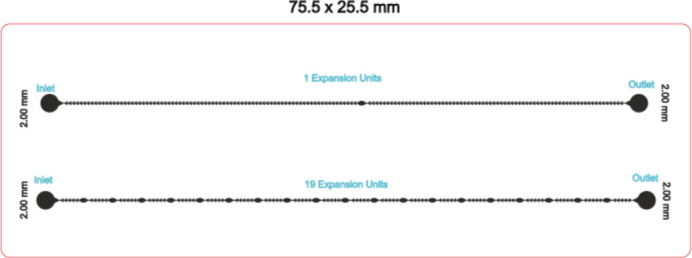
Schematic diagram of chip A.

The first chip (labeled as **chip A**) consists of two channels with different numbers of expansion units. One channel has one expansion unit, and the other has 19 expansion units, both at the same length of 62.76 mm. The inlet and outlet holes are 2.00 mm in diameter. Figure 1 represents the diagram of the **chip A**.

**Fig.2:**
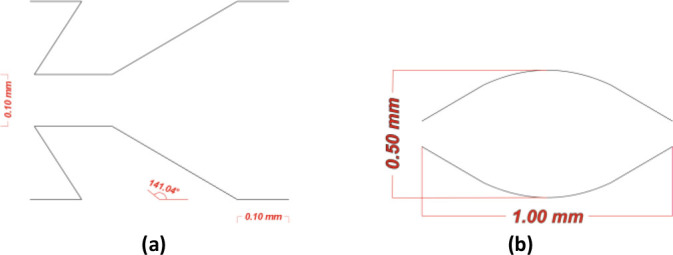
Detailed schematic diagram of the jagged teeth and expansion unit. (a) Jagged teeth dimensions, (b) The expansion unit dimensions.

The jagged surface must have an angle against the flow direction to remove the cumulus cells from the oocytes. The jagged surface mechanically removes the cumulus cells without needing enzymatic action like hyaluronidase. It applies direct shear stress on the oocyte to detach the COCs. The microfluidic chip ensures complete denudation without manual handling and operator variability. According to the literature, the best angle to remove the COCs is almost 141° [35]. Figure 2 (a) shows the teeth angle; the inner width is 0.1 mm, and the distance between each tooth is 0.1 mm.

The expansion units’ effect is crucial. They improve fluid dynamics for soft cell processing and facilitate a more effortless transfer of oocytes through the chip. They also optimize the shear force upon the oocyte to remove the COCs and avoid destroying the oocytes. The expansion units will guarantee that all faces of the oocytes are exposed to the jagged surfaces. Figure 2 (b) describes the dimensions of the expansion units. The maximum height (thickness) is 0.5 mm, and the maximum width is 1.00 mm. The chip has a constant depth of 0.1 mm.

The key motivation behind rendering **Chip A** is to allow for a direct comparison of minimal versus extensive expansion unit implementations, ensuring optimized shear stress application. The impact of expansion units will be studied by increasing the number of expansion units and the microfluidic channel length while maintaining the same width and depth. This might be considered a case study.

**Fig.3:**
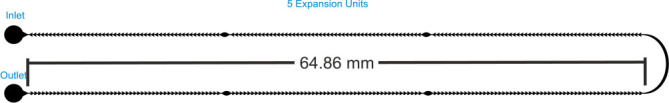
Schematic diagram of chip B.

The second chip (labeled as **chip B**) comprises two lines measuring 64.86 mm, with five expansion units. Figure 3 shows the second microfluidic channel. The connection between the upper and lower channels is considered an expansion unit with a larger space because the thickness of this connection channel is 0.5 mm. The motivation in studying the configuration of **Chip B** is to outline the performance frontiers of a multi-line design, assessing how interconnected expansion units influence oocyte transport and denudation.

**Fig.4:**
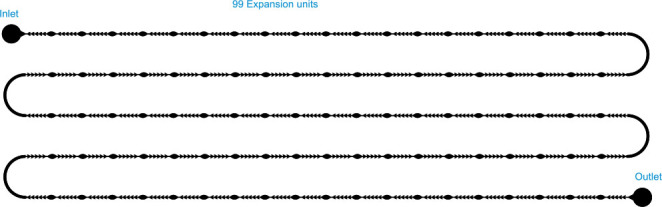
Schematic diagram of the third denudation chip

In line with the above configurations, another study increased the number of expansion units and channel lines, labeled as **chip C**. Figure 4 describes the third microfluidic **chip C** with 99 expansion units and five channel lines. The connection channels are also considered expansion units.

The key motivation in including **chip C** in our study is to further increase the complexity of the configuration by involving multiple expansion units and channel lines, enabling a comprehensive analysis of fluid dynamics.

The above-mentioned three chips will undergo CFD simulation to evaluate the effect of shear stress and real lab experimentation using actual buffalo oocytes to assess the denudation efficiency, depending on the number of expansion units at the channel length. All chips possess a uniform depth and an interior teeth width of 0.1 mm. The mean angle of all teeth across all chips is 141°.

### Fabrication Methodology

**Fig.5:**
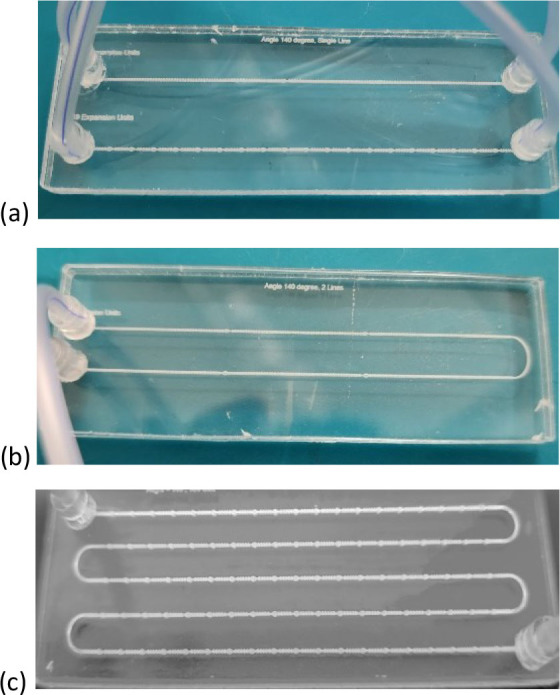
The Fabricated Chips with the connected tubes. (a) Chip A. (b) Chip B. (c) Chip C.

The fabrication utilized a CO_2_ laser machine (VLS3.5, Universal Laser Systems, Kanagawa, Japan) with a 30-watt laser tube. A laser lens (HPDFO, Universal Laser Systems, Japan) with a 30 µm focal spot was utilized. Microfluidic chips were fabricated using PMMA sheets that were 3 mm thick for the top and bottom layers and 0.1 mm thick for the design layer. To achieve high-quality results, the laser beam for patterning PMMA sheets was adjusted to 1000 pulses per inch (PPI). The laser power was set to 30% and the speed to 40% to achieve the optimal dimensions as specified in the design. The chip is composed of three layers of PMMA. The chip design was executed using CorelDraw ×5. The design was thoroughly engraved on the middle layer, whereas the inlet and outlet were drilled into the top layer. The three layers were thermally adjusted and bonded in a natural ventilation laboratory oven (STF-N 80, FALC Instruments, Treviglio, Italy) for 13 minutes at 150 °C under a force of 2 Newton. Subsequently, connection tubes with an outer diameter of 1.8 mm (ULTRAMED, Assiut, Egypt) were attached and secured at the inlet and outlet openings. The tube has an outer diameter of 1.8 mm, while the inlet and outlet holes measure 2 mm. Consequently, a PMMA cylinder with an outer diameter of 2 mm and an inlet diameter of 1.8 mm was incorporated to connect the tubes to the holes, ensuring a leak-free connection. Figure 5 shows the actual fabricated chips.

After the middle layer was fabricated, the channel width was measured using a Fluorescent Trinocular Compound Microscope - AmScope (FMSYG580TA) Supplies 40x–1600x Infinity Plan EPI. The advantage of the three-layer fabrication is that it ensures channel depth. After bonding the chip, further measurements using the same method were performed to confirm the dimensions’ stability. Our methods achieved high dimensional stability after fabrication and bonding, almost matching the design. A syringe pump (NE-4000 Pump Systems Inc.) was used to inject deionized (DI) water into the chip from the input to test for leakage. There was absolutely zero leakage consequently. To test and study the thermal effect of the bonding technique, a higher bonding force was applied for 13 minutes at 150 degrees Celsius, which is lower than the melting temperature of PMMA. Chip deformations and some changes in dimensions were noticed. On the other hand, when applying a lower bonding force, air bubbles and leakage were obtained. Therefore, the optimal condition is using the force at 2 Newton at 150 degrees Celsius for 13 minutes in a natural ventilation lab oven to achieve a high transparency chip with no leakage and suitable dimensions compared to the design.

### Preparation of the Oocytes

**Fig.6:**
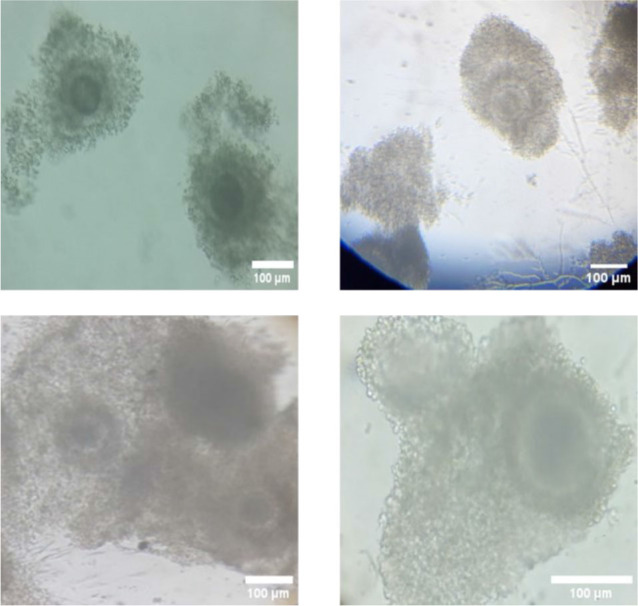
Different shots of the Mature oocytes with cumulus cells under the microscope.

The ovaries of female slaughtered buffalo are obtained from the Dachlout abattoir in Dayrout, Assiut Governorate, which are then transported to the Veterinary Medicine department at Assiut University to undergo washing and cleaning procedures to facilitate the extraction of oocytes. The process is essential as it involves the extraction of oocytes from the ovaries while maintaining integrity. Subsequently, the oocytes are placed in Tissue Culture Medium 199 (TCM 199) (BIO-CHEM, Belgium) and some other materials with specific concentrations for preservation and maturation, as described in Table 1. During the maturation process, oocytes in media were placed in small tubes within a CO_2_ incubator (N-BIOTEK Co, Korea) set at 38 degrees Celsius with 5% CO_2_ for a duration of 22 to 24 hours, preparing them for the subsequent denudation process. Figure 6 shows the oocytes after maturation (metaphase II) with the cumulus cells around them, which harden fertilization. Although the cumulus cells harden the fertilization, the COCs around the oocytes are vital because they regulate oocyte maturation and meiotic resumption [59]. It also creates metabolic cooperation between the COCs and the oocytes [60] and intercellular communication within the COC is crucial for oocyte development [61].

**Table 1:** The maturation media concentrations [62].

**Component**	**Final Concentration**
TCM 199	9 ml
Fetal Bovine Serum	1 ml
Amphotericin B	1 ml
FSH	10 µl
Sodium Pyruvate	20 µl
Gentamycin	10 µl
Estradiol 17β	10 µl

### Mathematical Equations

The primary aspect to consider is the wall shear stress cumulus oocyte cells will encounter when subjected to the jagged units that eliminate all cumulus corona cell mass. The flow rate must be adequate to eliminate cumulus corona cell mass while ensuring the preservation of oocytes, as shear stress is directly proportional to the flow rate. Microfluidics can be modeled mathematically. The most commonly used mathematical models in microfluidics are based on the steady-state Navier–Stokes equation for a Newtonian fluid [63,64].

(1) $$\frac{\partial u}{\partial t}+(u\cdot \nabla )u-\frac{\mu }{\rho }\nabla^{2}u=-\frac{1}{\rho }\nabla p+g$$

where *u* is the normalized flow velocity vector, *p* is the pressure, *t* is the time, *ρ* is the fluid’s density, *∇* is the divergence, *µ* is the dynamic viscosity, and *g* is the gravitational constant. In microfluidic systems, surface forces outweigh volume forces like gravity [65]. Therefore, gravitational force may be ignored. Furthermore, the fluid is incompressible, which removes velocity gradients within the fluid volume [66]. With these assumptions, equation (1) can be simplified to the following:

(2) $$\frac{\partial u}{\partial t}-\frac{\mu }{\rho }\nabla^{2}u=-\frac{1}{\rho }\nabla p$$

An analytical solution for velocity profiles in rectangular-based microfluidic channels was found by expanding the Navier-Stokes equation using the Fourier series [67]. However, in simple geometry, such as a wide rectangular or tubular channel, computational fluid dynamics was utilized to determine the wall shear stress as a function of the channel width and flow rate. Mathematically, the wall shear stress can be calculated in rectangular channels using the following equation [68,69].

(3) $$\tau =\frac{6\eta Q}{h^{2}w}$$

where *h* is the channel height, *w* is the channel width, *Q* is the flow rate, and *η* is the medium viscosity. The viscosity of the TCM 199 medium with other mediums is 0.0011 Pa·s (Pascal Second). The velocity can be calculated using the following equation:

(4) Q=A⋅v

where *A* is the area, and *v* is the velocity. We inject the fluid constantly, but the velocity will change inside the microfluidic channel. Therefore, the shear stress changed at each point, and consequently, Computational Fluid Dynamics (CFD) is required to calculate the parameters at each point after meshing analysis.

### Computational Fluid Dynamics (CFD) Simulation of Velocity and Shear Stress

**Fig.7:**
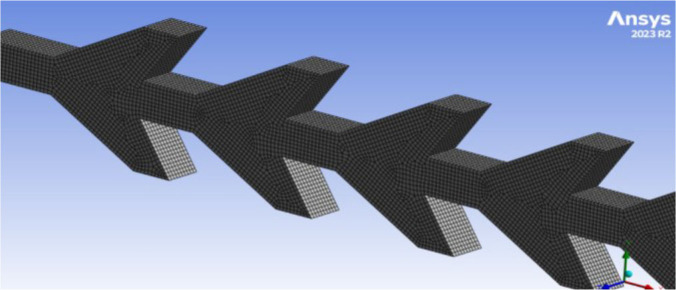
Meshing analysis in Ansys.

A three-dimensional (3D) model of each design in the microfluidic chips was conducted on Ansys Fluent 2023 R2 (ANSYS, Inc., Canonsburg, PA) to evaluate and calculate the wall shear stress affected on the oocytes. The geometry was constructed by drawing our design with accurate lengths and widths on the geometry part in the Ansys Fluent project. Then, the design was extruded with a 0.05 mm depth in both-symmetric to get 0.1 mm depth. The design was established as an actual chip design. A named selection was created in the meshing part to specify the inlet, outlet, fluid domain, and wall. The wall is assumed to be the whole design without the inlet and outlet. Then, geometry and material were selected, and an automatic method for meshing was used. The element size was selected and then reduced more and more until the same output results were obtained. After many trials, an element size of 10 μm in the body sizing analysis was found enough to ensure high accuracy without changing the output results if we reduce it. Figure 7 shows the meshing analysis in Ansys, specifying the element size.

Double precision is selected in the setup and solution section, the time is steady, and a laminar flow (due to the Reynold number) is selected with specified fluid parameters such as viscosity. The viscosity of the TCM medium with other mediums is 0.0011 Pa·S (Pascal Second). The flow rate will be changed to see the shear stress effect.

### Experimental Denudation Process

After preparing the oocytes, 0.5 mL of media with about 15 oocytes was transferred to an Eppendorf tube. A micro-pipette to manipulate the 0.5 mL volume is essential to avoid oocyte adhesion to the tube’s walls or bottom. The pumping technique employed in this context is distinct. If fluid is pumped from the inlet to the outlet, it will remain in the tube and not enter the microfluidic chip. Consequently, an alternative pumping technique was employed. The output tube was connected to a 10 mL sterile syringe (Jiangxi Hongda Medical Equipment Group, Nanchang, China) and placed into a syringe pump (NE-4000, New Era Systems, Grant, FL, USA). In comparison, the input tube was inserted into the micro-Eppendorf tube. Subsequently, the flow rate was adjusted. The withdrawal operation is initiated to extract the fluid from the reaction tube, traverse the designated channel, and collect the fluid in the syringe.

All fluid was rapidly injected through the channel, ensuring no fluid remained in the input tube and the input tube was not removed from the syringe, nor was air infused to facilitate fluid movement to the channel. The injected fluid was subsequently stored in the medium syringe that contained the denudated oocytes. To determine the output results, an MT5000(IFR) 5.0MP CCD fluorescent microscope camera, which has a Sony ICX282AQ sensor, was mounted on the Fluorescent Trinocular Compound Microscope – AmScope (FMSYG580TA). The microscope and the camera are connected to the lab computer to see the results.

### Ethical approval

The conducted research is not related to either human or animal use.

## Results

### Computational Fluid Dynamics (CFD) Results

**Fig.8:**
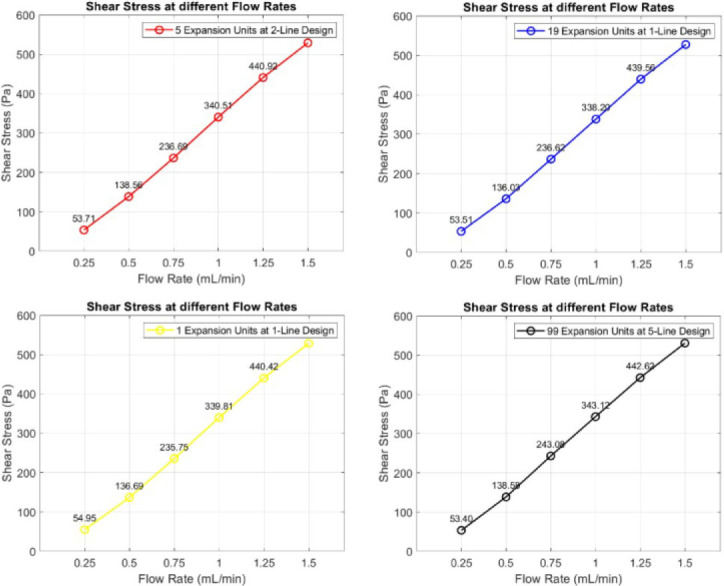
Shear stress at each design’s first pair of teeth at different flow rates.

In the Ansys results section, the wall shear stress was studied. The maximum shear stress occurs at the beginning of the first pair of teeth. Different flow rates were applied to evaluate the effect of varying the number of expansion units upon the shear stress. Figure 8 describes the maximum shear stress applied to each design’s first pair of teeth at different flow rates, such as 0.25, 0.5, 0.75, 1, 1.25, and 1.5 mL/min.

**Fig.9:**
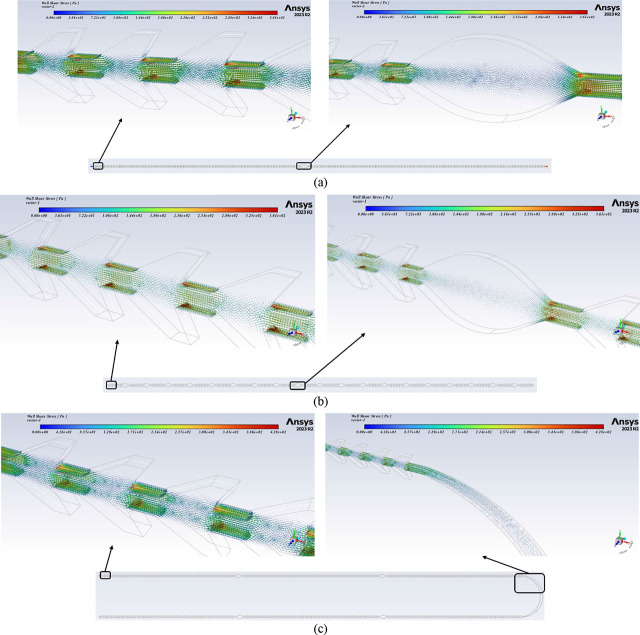

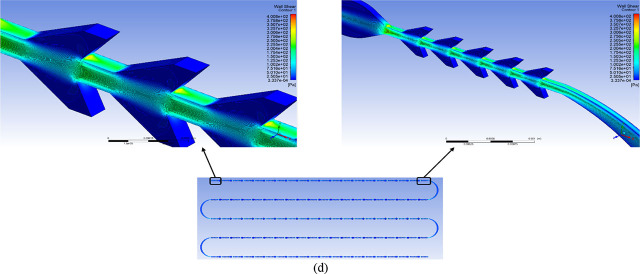
The simulation results of the chips on Ansys. (a) The first channel of chip A is “1 expansion unit at 1 line design”. (b) The second channel of chip A is “19 expansion units at 1 line design”. (c) Chip B is “5 expansion units at 2-line design”. (d) Chip C is “99 expansion units at 5-line design”.

Figure 8 illustrates a negligible difference across the designs, indicating that various numbers of expansion units do not influence the shear stress exerted on the oocytes. These chips will undergo testing with actual oocytes in the laboratory to evaluate denudation efficiency. Figure 9 (a), (b), (c), and (d) present the CFD simulation results (vector or contour) for the three chips.

### Experimental Results

**Fig.10:**
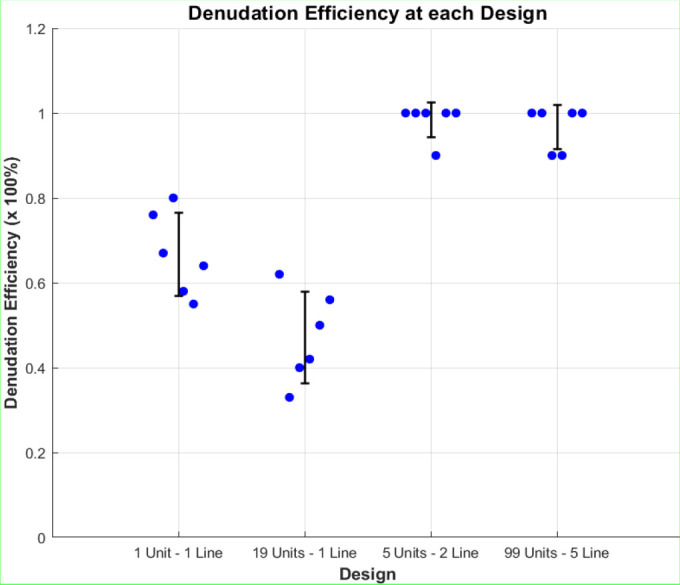
Denudation efficiency at the three chips. Where the first two channels belong to chip A, the third channel belongs to chip B, and the fourth channel belongs to chip C.

Following the analysis of simulation results, empirical laboratory experiments are performed to ascertain the appropriate design and effectiveness of modifying the expansion units in relation to denudation efficiency. Denudation efficiency refers to the ratio of denudated oocytes to the total number of (output) oocytes. Simulation results indicate that shear stress remains nearly uniform across all chip designs. Denudation efficiency will be calculated for each chip design. Figure 10 illustrates the denudation efficiency associated with each chip design. About 15 oocytes have been used at each group trial.

As seen in Figure 10, the highest denudation efficiency occurs at chip B and chip C. Chip B consists of 5 expansion units at a 2-line design and achieved almost 98.33% denudation efficiency. Chip C comprises 99 expansion units at a 5-line design, achieving almost 96.67% denudation efficiency. Efficiency decreased dramatically in chip A, reaching 66.67% at the first and 41.17% at the second channel. Error bars represent Standard Deviation (SD), which measures how much individual data points deviate from the mean of the dataset.

## Discussion

By analyzing the results presented in Figures 8, 9, and 10, we can conclude that shear stress is crucial for denudation because it facilitates the mechanical removal of cumulus cells from the oocyte surface, reducing the need for enzymatic treatments. However, the number of jagged surfaces the oocytes encounter is a more influential factor, as it directly determines the extent of mechanical interaction and the effectiveness of cumulus cell detachment. To optimize the denudation process, oocytes must be exposed to an adequate number of jagged surfaces for efficient cumulus cell removal, while a sufficient number of expansion units is necessary to promote controlled oocyte rotation. This rotation ensures that all surfaces of the oocytes interact with the jagged structures, enhancing shear exposure and improving fluid dynamics within the microfluidic chip, ultimately leading to higher denudation efficiency.

**Fig.11:**
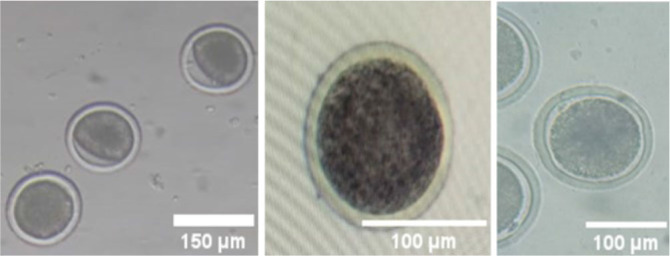
Shots of the complete denudated oocytes.

When the flow rate decreases, the wall shear stress will be reduced, and the denudation efficiency will be reduced. If the flow rate increases, the output of oocytes will be reduced due to destruction. The optimal design that achieves the highest denudation efficiency without using enzymatic action like hyaluronidase is the five expansion units at a 2-line design (one line measuring about 64.8 mm) at a constant flow rate of 1 mL/min. Figure 11 shows different shots of the complete denudated oocytes. Most oocytes show the polar body, indicating the perfect maturation and denudation process. Recent studies suggest that polar bodies play a role in embryonic development, and their form correlates with embryo quality and potential [70].

**Fig.12:**
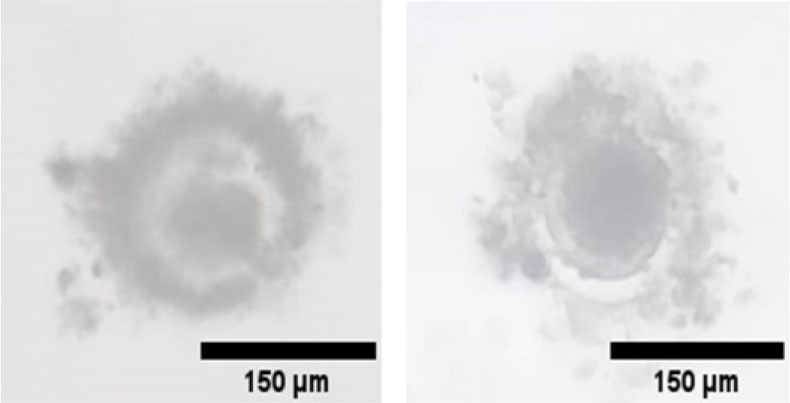
Shots of the incomplete denudated oocytes.

Figure 12 illustrates the incomplete denudated oocytes due to low wall shear stress or insufficient exposure to jagged surface units. As noted, cumulus corona cells remain around the oocytes, which can decrease the fertilization rate and harden the IVF of the ICSI process.

**Fig.13:**
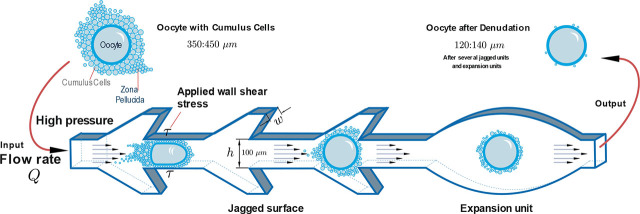
The mechanical concept of the microfluidic chips.

Figure 13 illustrates the mechanical interaction between the jagged surfaces, expansion units, and oocytes within the microfluidic channels. The input flow remains laminar, transporting un-denuded oocytes through the system. As the oocytes progress through the microfluidic chip, they encounter jagged surfaces, which generate localized shear stress to facilitate cumulus cell removal. Simultaneously, expansion units regulate the flow dynamics, promoting controlled oocyte rotation and ensuring uniform exposure to the shear forces. At the outlet, the oocytes emerge fully denuded, demonstrating the effectiveness of the microfluidic design in achieving efficient and consistent oocyte processing.

## Conclusion

This study involved the design and manufacturing of microfluidic chips utilizing design software to test the effect of varying the number of expansion units in microfluidic chips for oocyte denudation, followed by the fabrication through direct CO_2_ laser engraving on PMMA material to reduce the fabrication cost. The channels were divided into jagged surface units that remove the cumulus cells around the oocytes and expansion units to enhance fluid dynamics for soft cell processing. The configuration facilitates the transfer of oocytes through the chip, optimizes shear force to remove the cumulus-oocyte complexes without damaging the oocytes, and ensures that all surfaces of the oocytes are adequately exposed to the jagged surfaces. The PMMA material has demonstrated its suitability and efficiency in medical applications. Three chips with four designs have been designed and manufactured to test each design’s shear stress and denudation efficiency. The chips consist of 3 layers; the middle layer thickness is 0.1 mm to ensure that the depth is constant through all chips. The chips’ three layers were adjusted and thermally bonded in a natural ventilation lab oven at 150 degrees Celsius for 13 minutes under the force of 2 N. The fabrication duration was about 20 minutes, while the fabrication cost was about 1 USD. A Computational Fluid Dynamics (CFD) simulation was conducted using ANSYS Fluent 2023 R2. The process involved designing the model, applying mesh analysis with an element size of 10 μm, executing the setup and solution phases, and rendering the results of wall shear stress. Almost the same level of wall shear stress was achieved at different chip designs while maintaining the tilting angle and flow rate. Therefore, real experimentation became essential to examine the impact of varying expansion units.

Different denudation efficiencies were obtained through the experimentation, and we found that chip B achieved 98.33% denudation efficiency, chip C achieved 96.67% denudation efficiency, the first channel in chip A achieved 66.67% denudation efficiency, and the second channel of chip A achieved 41.17%. The highest denudation efficiency at 98.33%, characterized by low cost and minimal fabrication time, was achieved with a design comprising five expansion units arranged in a 2-line design. This configuration is the best found among the existing ones because the oocytes are exposed to a reasonable number of continuous jagged surfaces and expansion units.

In the future work, it would be desirable to develop a develop a framework to automatically identify the efficiency of the oocyte output from the chips; as such the exploration of vision-based oocyte recognition and segmentation would facilitate the overall denudation process. We also plan to enable a low-cost actuation mechanism for an automated device in oocyte denudation schemes without using a syringe pump or related equipment to facilitate the denudation process.
